# Unrealistic energy and materials requirement for direct air capture in deep mitigation pathways

**DOI:** 10.1038/s41467-020-17203-7

**Published:** 2020-07-03

**Authors:** Sudipta Chatterjee, Kuo-Wei Huang

**Affiliations:** KAUST Catalysis Center and Division of Physical Sciences & Engineering, King Abdullah University of Science and Technology, Thuwal, 23955-6900 Saudi Arabia

**Keywords:** Energy, Energy policy

Arising from G. Realmonte et al. *Nature Communications* 10.1038/s41467-019-10842-5 (2019).

The increasing global atmospheric CO_2_ concentration due to heavy reliance on fossil fuels as the primary energy sources (~410 ppm in 2019)^1^ has made direct extraction or removal of CO_2_ from ambient air (direct air carbon capture (DACC)) the most logical alternative over traditional modes of carbon capture from large stationary sources because of many of the perceived advantages and compelling arguments^2^. With the current level of CO_2_ emissions (32.6 gigatons (Gt)-CO_2_/year_2017_)^1^, Realmonte and co-workers recently imposed the global capacity at 30 Gt-CO_2_/year as a case study for DACC, and concluded that “in theory DACCS can be an enabling factor for the Paris Agreement objectives” and recommended the policy makers to “support an acceleration in development and deployment of DACCS”^3^. While challenges of large-scale CO_2_ utilization and sequestration were recognized and these approaches were deemed impractical^4,5^, our analysis further showed that the energy and materials requirements for DACC are unrealistic even when the most promising technologies are employed. Thus, DACC is unfortunately only an energetically and financially costly distraction in effective mitigation of climate changes at a meaningful scale before we achieve the status of a significant surplus of carbon-neutral/low-carbon energy.

The urgent need for a large amount of CO_2_ removal by DACC has been recognized since the Intergovernmental Panel on Climate Change (IPCC)’s assessment report in 2013^6^. This rapidly growing field has thus attracted industrial, academic and political attention over the last decades, ultimately pushing DACC technologies from lab scale to pilot scale with multi-billion dollars of investments and research funding from the private and governmental sectors^7^. Realmonte et al. have investigated the roles of DACC utilizing two integrated assessment modelling (IAM) studies on DAC1 (based on aqueous hydroxide solutions of NaOH, KOH, etc.) and DAC2 (based on amine-modified solid sorbents such as monoethanolamine (MEA)) as the most promising methods (Fig. 1). Detailed techno-economic characteristics have been incorporated based on the latest available literature estimates aiming to meet the Paris Agreement objectives to keep global warming well below 2 °C (i.e. removal of 30 Gt-CO_2_/year). However, to fully assess the feasibilities of DACC technologies, the energy cost for manufacturing materials must be considered.

**Fig. 1:**
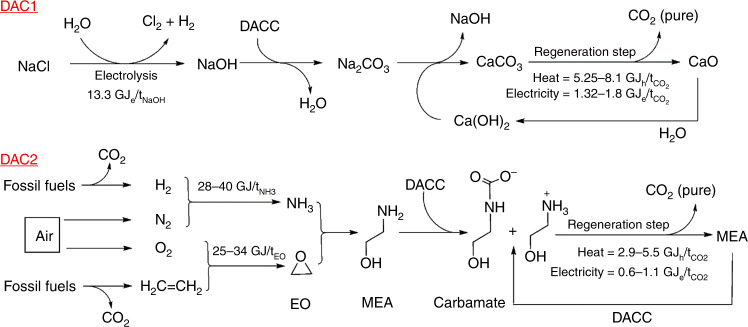
Schematic representations of direct CO_2_ capture from air using sorbents. The outlines of two different DACC technologies (DAC1 and DAC2) are presented with the amounts of electricity and heat energies required for the syntheses of the sorbents (NaOH and MEA) from precursors and their regenerations^3^. DACC direct air carbon capture, EO ethylene oxide, MEA monoethanolamine.

The total energy requirement to reach the capacity of capturing 30 Gt-CO_2_/yr was estimated based on the production of the needed materials as reasonably proposed by Realmonte et al. considering the make-up rates of sorbents of 0.17–0.29 t/t_CO2_ of NaOH in DAC1^8^ and a similar replacement rate for MEA in DAC2. For DAC1, 5.1–8.7 Gt/yr of NaOH is required, and the production will need 2.15–3.67 TW_e_-yr (electrical energy for electrolysis = 13.3 GJ_e_/t_NaOH_)^9^ (Fig. 2a, b). This will account for about 12–20% of total global energy supply (TGES; 18.55 TW-yr for 2017, but likely greater than the global electricity generation capacity of 2.92 TW_e_-yr)^10^. One also notes that the industrial electrolysis process, associated with NaOH production in DAC1, will result in the production of a huge amount of Cl_2_ gas (4.6–7.9 Gt), far exceeding the current utilization capacity (76.8 Mt Cl_2_/yr) and posing risks which are difficult to evaluate a priori. In addition to the materials production energy cost, sorbents regeneration also drag a significant amount of heat and electricity. While DAC1 employs the less “energy-intensive” NaOH, the necessity of high temperature (>800 °C) for regeneration induces a larger energy usage (6.57–9.9 GJ_total_/t_CO2_ (1.32–1.8 GJ_e_/t_CO2_ and 5.25–8.1 GJ_h_/t_CO2_), 6.25–9.41 TW_total_-yr = 34–51% TGES) (Fig. 2c). In contrast, even though the milder condition for sorbent regeneration in the DAC2 technology costs ~50% of that for DAC1 (3.5–6.6 GJ_total_/t_CO2_ (0.6–1.1 GJ_e_/t_CO2_ and 2.9–5.5 GJ_h_/t_CO2_), 3.3–6.3 TW-yr = 18–34% TGES)^11,12^, 16.3–27.8 Gt of NH_3_ and 3.3–5.6 Gt of ethylene oxide (EO) will be necessary to produce the required amount of MEA (i.e. 5.1–8.7 Gt), which will consume 14.5–24.7 TW_h_-yr (28 GJ_h_/t_NH3_ if produced only from natural gas^13^, 78–133% TGES) or 20.7–35.3 TW_e_-yr (40 GJ_e_/t_NH3_ if produced only by electrolysis^13^, 112–190% TGES) and 2.6–4.1 TW_total_-yr (25 GJ_total_/t_EO_, 14–24% TGES)^14^, respectively (Fig. 2).

**Fig. 2:**
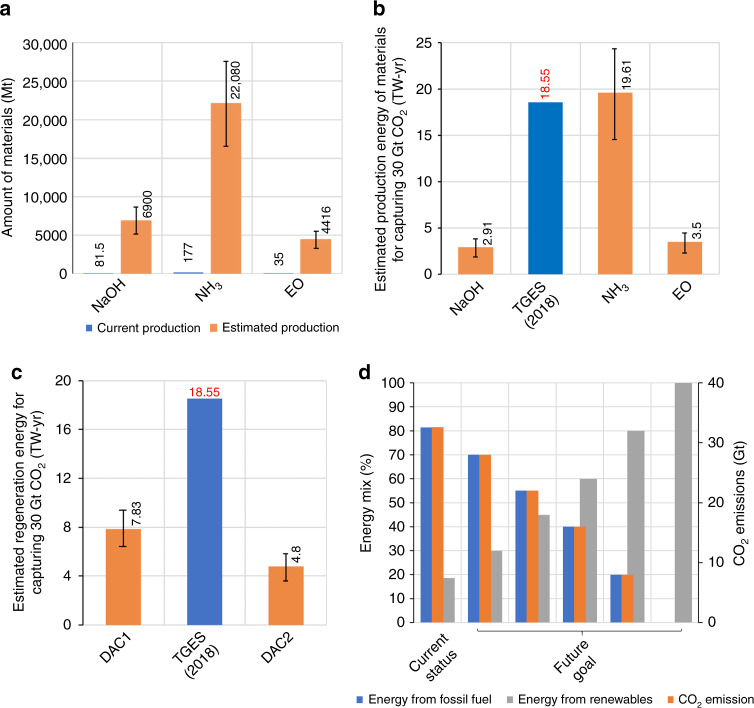
Energy and materials requirement for DACC—current status and prospects. **a** Estimated materials required for CO_2_ (30 Gt) capture in DACC and compared to their current production. Estimated energy required for **b** material production and **c** sorbent regeneration. **d** Current and future energy mix with concomitant CO_2_ emissions.

From our analyses, DAC2 is clearly an unsuitable option to capture 30 Gt-CO_2_/yr, most likely due to enormous amount of materials (16.3–27.8 Gt of NH_3_ and 3.3–5.6 Gt of EO) and energy needed (20.4–35.1 TW-yr, 110–191% TGES if NH_3_ production from only natural gas is being considered). DAC1 also takes at least 8.4–13.1 TW-yr (46–71% TGES), excluding the potential environmental risks and the associated energy costs required for carbon storage and utilization (CSU)^4^. Since the replacement of every 10% TGES under the current energy status by low-carbon sources, such as nuclear, hydro, wind and solar, can reduce ~4 Gt-CO_2_/yr of emission (Fig. 2d), it is not logical to allocate low-carbon energy for DACC at present without accelerating the depletion rate of limited fossil fuels.

Considering the projected global energy demand to be increased at least by 50% before 2050^15^, it is imperative that TGES cannot only be invested to harness the CO_2_ issue. While DACC (and CSU) technologies are an important topic of R&D and may indeed offer some commercial opportunities with incentivized carbon prices (given the surplus electricity is available), before a significant amount of carbon-neutral and/or low-carbon energy can be deployed, any DACC activities will be a significant distraction with negligible contributions to mitigating climate changes. Concentrated efforts on the deployment of low-carbon energy generation plants across the globe together with raising public awareness to use alternative energy and on the development of technologies in enhancing the energy usage efficiency (e.g. in transportation) must be prioritized to address the pressing twin problems of climate changes and energy security.

Finally, discerning the number of related challenges in DACC and CSU^4,5^, it is of utmost importance to conduct not only a thorough techno-economic analysis on the future proposed processes but comprehensive assessments on the materials and energy needs at the scale of multi-Gt-CO_2_/yr before a realistic solution can be identified. Wide-ranging discussions/debates between experts from all sectors from all angles are urgently needed.

## Data Availability

All relevant data supporting the findings of this study are available within the paper.
